# Evaluation of Dimer of Epicatechin from an Endophytic Fungus *Curvularia australiensis* FC2AP on Acute Toxicity Levels, Anti-Inflammatory and Anti-Cervical Cancer Activity in Animal Models

**DOI:** 10.3390/molecules26030654

**Published:** 2021-01-27

**Authors:** Vellingiri Manon Mani, Arockiam Jeyasundar Parimala Gnana Soundari, Balamuralikrishnan Balasubramanian, Sungkwon Park, Utthapon Issara, Kathirvel Preethi, Wen-Chao Liu

**Affiliations:** 1 Department of Biotechnology, Rathnavel Subramaniam College of Arts and Science, Coimbatore, Tamil Nadu 641402, India; manonmanisathee12@gmail.com; 2 Department of Microbial Biotechnology, Bharathiar University, Coimbatore-46, Tamil Nadu 641046, India; apgs_1987@yahoo.co.in; 3 Department of Environmental Science & Engineering, College of Natural Resources & Environment, Northwest A & F University, Xianyang 712100, China; 4 Department of Food Science and Biotechnology, College of Life Science, Sejong University, Seoul 05006, Korea; sungkwonpark@sejong.ac.kr; 5 Division of Food Science and Technology Management, Faculty of Science and Technology, Rajamangala University of Technology Thanyaburi, Pathum Thani 12110, Thailand; utthapon_i@rmutt.ac.th; 6 Department of Animal Science, College of Agriculture, Guangdong Ocean University, Zhanjiang 524088, China

**Keywords:** acute toxicity, albino mice, anti-cancer, anti-inflammatory, cervical cancer, dimer of epicatechin, metabolites, Sprague-Dawley rats

## Abstract

Cervical cancer, as the most frequent cancer in women globally and accounts almost 14% in India. It can be prevented or treated with vaccines, radiation, chemotherapy, and brachytherapy. The chemotherapeutic agents cause adverse post effects by the destruction of the neighboring normal cells or altering the properties of the cells. In order to reduce the severity of the side effects caused by the chemically synthesized therapeutic agents, the current research developed an anti-cancer agent dimer of epicatechin (DoE), a natural bioactive secondary metabolite (BSM) mediated from an endophytic fungus *Curvularia australiensis* FC2AP. The investigation has initiated with the evaluation of inhibiting the angiogenesis which is a main activity in metastasis, and it was assessed through Hen’s Egg Test on Chorio Allantoic Membrane (HET-CAM) test; the BSM inhibited the growth of blood vessels in the developing chick embryo. Further the DoE was evaluated for its acute toxicity levels in albino mice, whereas the survival dose was found to be 1250 mg/kg and the lethal dose was 1500 mg/kg body weight of albino mice; hematological, biochemical, and histopathological analyses were assessed. The anti-inflammatory responses of the DoE were evaluated in carrageenan induced Wistar rats and the reduction of inflammation occurred in a dose-dependent manner. By fixing the effective dose for anti-inflammation analysis, the DoE was taken for the anti-cervical cancer analysis in benzo (a) pyrene induced female Sprague-Dawley rats for 60 days trial. After the stipulated days, the rats were taken for hematological antioxidants, lipid peroxidation (LPO), member bound enzymes, cervical histopathological and carcinogenic markers analyses. The results specified that the DoE has the capability of reducing the tumor in an efficient way. This is the first report of flavonoid-DoE production from an endophytic fungus *C. australiensis* has the anticancer potentiality and it can be stated as anti-cancer drug.

## 1. Introduction

Cervical cancer is the most malignant cancer in the women ranking second globally and it affects the women aged from 35 to 60 years [1]. Majorly it was caused by human papilloma virus (HPV) infections such as high risk and low risk HPV infections. Apart from HPV infections still there are many other factors, such as history of genital warts, AIDS patients, tobacco smoking, and multiple sexual partners, which play their role in inducing the cervical cancer and lesions [2,3]. The treatment for cervical cancer begins with chemotherapy, surgery, and radiotherapy; these therapies remains to be the major strategies in treating not only cervical cancer but also other types of cancer. The chemotherapy has certain extent in saving many lives but still this therapy produces some immunological ill effects in the patients [4]. The chemotherapy includes the chemical or artificial medicinal agents which alters and damages the DNA in the tumor cells which reduces the ill-effect of cancer. These side effects could be reduced or prevented by the implementation of natural cancer therapeutic agents. The natural bioactive agents or products can be extracted from plants, algal, and microbial sources. The Indian traditional medicinal system has several medicines which can treat various types of diseases and disorders using medicinal plants and trees. However, it is not possible to cut down or damage the plants/trees for retrieving the medicines, as for treating a single patient it is necessary to take at least 1 kg of the plant sources. So the current research has aimed to isolate an endophytic fungi residing in the medicinal tree that could possess the metabolic nature of the host tree and aids in the production of the expected bioactive cancer therapeutic agent from a microbe which could be produced maximum quantity in a minimum culture area.

The endophytic fungi contain a broad classification of bioactive secondary metabolites (BSMs) with distinctive nature including alkaloids, flavonoids, phenols/polyphenols, benzopyranones, quinones, chinones, terpenoids, steroids, tetralones, and xanthones [5]. Most of the BSMs are effectively used in the medicinal field as cancer therapeutic agents which prevented/treated successfully. Wu et al. [6] explored that the anti-cancer activity exhibited by the potential endophytic fungal strains in breast cancer cell lines. The strains of *Aspergillus* produced the anti-tumor compounds and explored its therapeutic properties in inhibiting the growth of MCF-7 cells [7,8]. Majoumouo et al. [9] has reported that the potential anti-cervical cancer properties in metabolites extracted from the endophytic fungal strains such as *Fusarium*, *Phoma*, and *Colletotrichum* species. He et al. [4] reported that the anti-cancer activities of BSMs from the endophytic fungi and the BSMs explicated the anti-cervical cancer activity efficiently in animal models. On comparing to other therapeutic metabolites epicatechins and its derivatives were found to exhibit the highest cancer therapeutic properties especially in treating and preventing the cervical cancer [10]. These epicatechins and its derivatives have been reported to exhibit the anti-tumor activities by the inactivation of tumor metabolic pathways in various kinds of cancer. The epicatechins derivatives inhibits the activation of STAT3 pathway which has a significant role in the formation of tumor, and this was reported in nasopharyngeal carcinoma [11]. Few researches supports the anti-cancer activities explicated by the epicatechin derivatives in the prevention and decrease of tumor formation, progression, and metastasis stages [12,13,14,15]. These epicatechins are also helpful in the prevention and curing of neurodegenerative diseases [16]. Elbaz et al. [17] reported the anti-pancreatic cancer activity exhibited by the epicatechins where the metabolites stimulated the mitochondrial activity upon radiation exposure. Moreover, the epicatechins are a good anti-proliferative agents in breast cancer cells when there are bio catalytically oligomerized [18].These epicatechins family are highly present in tea plant but this epicatechin derivatives was remarkably present in an endophytic fungus *Curvularia australiensis*. The presence of epicatechins in *C. australiensis* was from the metabolic process mimicked from the host tree *Aegle marmelos* [19]. The *Curvularia* species are found to contain more medically important metabolites and few research reports were found to explicate the results on potential metabolites. The *Curvularia* sp. was isolated from *Murraya koengii* was found to exhibit the inhibition of *Phytophthora capsica* zoospores [20]. Khiralla [21] reported that the cytotoxicity effects of metabolites produced by an endophytic fungus *C. papendroffi* and its effect on cancer cells. There are only few researches on the metabolites produced by the *C. australiensis* in medicinal fields. The goal of this current investigation was to evaluate the effects of dimer of epicatechin (DoE) on anti-angiogenesis, anti-inflammation, and anti-cervical cancer properties through in vitro and in vivo animal studies.

## 2. Methodology

### 2.1. Curvularia australiensis Strain and Source

This investigation has focused to characterize the anti-cancer potentiality of the DoE produced by an endophyte *C. australiensis* FC2AP. This strain was isolated from a medicinal tree *Aegle marmelos* grown around the foothills of Vellingiri hills, Western Ghats regions (Nilgiris cluster), Coimbatore, Tamil Nadu, India. The strain FC2AP (NCBI accession number: KR363626) was isolated from the leaf sample of the *A. marmelos* tree; the strain was found to dwell and produce maximum metabolites in Sabouraud’s Dextrose Broth medium [22]. The preliminary research of this study such as isolation, identification, and characteristics of the isolated endophytic fungus *C. australeinsis* FC2AP was stated in the earlier investigation of Mani et al. [22]. The DoE was purified through HPLC (High Performance Liquid Chromatography) technique from crude pigmented metabolites extract-CPME (biomass-intracellular extract) produced by *C. australiensis* in Sabouraud’s Dextrose Broth medium; this medium was optimized using experimental design- RSM executed through Stat-Ease software. There were six peaks (MM1 to MM6) when CPME was eluted at 554 nm and those eluted fractions were subjected to antimicrobial and antioxidant analyses where the highest peak (MM4) exhibited the maximum activity at minimum concentration. The MM4 was put through for chemical characterization of the structure with the aid of UV-Vis spectrum, FT-IR, 1H, and 13C NMR, and HRLC-MS/MS analyses. With these data, the compound MM4 was found to be DoE with the molecular mass of 573.1991 (C_30_H_22_O_12_). The elucidated DoE was investigated for anti-cervical cancer properties on cancer cell lines HeLa and the DoE explicated its anti-cancer potentiality where the cell viability in HeLa cells was found to be 48.38% at 100 µg/mL concentration (unpublished data).

### 2.2. In Vitro Study: Anti-Angiogenic Analysis

This analysis was executed by Hen’s Egg Test on Chorio Allantoic Membrane (HET- CAM), assessed to observe the inflammation/inhibition of tissue reactions by the BSM- DoE on the growing tissues and blood vessels [23]. The shell and capsular membrane of 9th day embryonated egg was carefully removed, and the DoE of 200 µL concentration was dispensed on small discs in triplicates and those discs were carefully inserted into the Chorio Allantoic Membrane. The number of blood vessels were counted at initial and after the incubation period of 2 and 18 h; this confirms the inhibition of angiogenic process. The positive control and negative control used in this evaluation were 0.1 N NaOH and 0.9% NaCl.

### 2.3. In Vivo Studies: Animal Models

The effectiveness of the DoE as a drug constituent has been tested out in the living biological system, the mice and rat models were prominent for checking the effect of compound DoE in in vivo analysis. The acceptance of new drug in the medicinal field is only after the drug has to be approved as non-toxic compound in the animal system. Hence the animal studies have been carried out using mice and rat as model systems. The animals used in this study were purchased from M/s. Venkateshwara Animal Breeders Pvt. Ltd., Bangalore, India. The animals were randomly selected and weighed to make certain of observance with the age invocated. The animals were accommodated in metabolic cages (size: 55(L) × 32.7(B) × 19(H) cm), with sawdust scraps, and each cage was accommodated with a maximum of 6 animals (same sex should be in a single cage).

All animals were kept in observation and acclimatization for a period of 20 date from the arrival date and the onset of treatment. The animals were examined by a veterinary physician to certify the fulfillment of health precondition for initiation of the research study. The rats were individually identified by the tattoos on the various parts of the body [Head (H), body (B), tail (T), head body (HB), body tail (BT), and no mark (NM)]. The marking of the animals was performed when the animals were distributed among the study groups. All animal procedures were performed in accordance with Institutional Animal Ethic Committee guidelines, after getting the approval from the Committee for the Purpose of Control and Supervision of Experiment on Animals at KMCH college of Pharmacy, Coimbatore, Tamil Nadu, India (IAEC No. KMCRET/Ph.D/05/2014-15).

#### 2.3.1. Acute Toxicity Study

##### Animal and Experimental Design

Acute oral toxicity was performed as per Organization for Economic Co-operation for Development guideline 423 methods. A total of sixty albino mice (8–10 weeks old with, initial average weight of 25–30 g; mixed gender) are fasted 6 h prior to dosing (food was withheld for 3 h but not water). Following the period of fasting, animals were weighed, and test substance DoE was administered orally in various concentration such as 2 g, 1.75 g, 1.5 g, 1.25 g, 1 g, 0.75 g, 0.5 g, 0.35 g, 0.25 g, and 0.1 g using specially designed mice oral needle. A total number of six animals were used for each dosage in each group and after the administration, food was withheld for 2 h. Animals were observed individually at least once during the first 1 h, periodically during the first 24 h, with special attention given during the first 4 h, and daily thereafter for 3 days, and for a total of 7 days. Animals were observed for their alertness, grooming, touch, torch and pain responses, tremors, convulsion, righting, pinna and corneal reflexes, gripping strength, pupils, urination, salivation, change in skin color, lacrimation and also for hyperactivity before and after administration of test samples. From these, the effective dosage and lethal dosage has been fixed.

##### Samples and Analysis

The animals were anaesthetized following the treatment period of 7 days, using ketamine hydrochloride and the blood was fetched from the animal’s retro-orbital sinus (three animals from each group). The collected blood was dispensed into the respective centrifuge tube containing an anti-coagulant-EDTA and further used hematological experiments. The hematological parameters like hemoglobin, Red Blood Cells (RBC), White Blood cells (WBC), Mean Corpuscular Volume (MCV), Packed Cell Volume (PCV), Mean Platelet Volume (MPV), differential cell count, Mean Corpuscular Hemoglobin Concentration (MCHC), and Mean Corpuscular Hemoglobin (MCH) count were determined [24,25,26,27]. The determination of biochemicals functioning, the separated serum from the collected blood sample was used and various biochemical parameters such as alkaline phosphatase (ALP), aspartate transaminase (AST), Alanine transaminase (ALT) [28,29], bilirubin, and total protein (TP) [30] were analyzed. Protein measurement was done using Folin’s phenol reagent.

Histopathological analysis reveals the pathological implications on tissues, and this was observed through microscopy. The tissues from various organs of healthy and dead animals were collected wither from biopsy or necropsy. Thin pieces of 3 to 5 mm thickness were sectioned, and the tissue(s) was kept in fixative (10% Formalin) for 24–48 h at room temperature. The section was deparaffinized using xylol for 5–10 min and excess xylol was removed by absolute alcohol. Then the section was cleaned and stained with hematoxylin for 3–4 min. The section was then counter stained with 0.5% eosin (15–30 s), excess stain was removed. The section was blotted, dehydrated in alcohol and cleared with xylol (15–30 s). It was then mounted on a Canada balsam or DPX Mountant. Further, the slide was kept dry and without air bubbles for future studies.

#### 2.3.2. Anti-Inflammatory Study

Female Wister albino rats weighed around 150 to 175 g were used for this study. The animals were segregated into five groups with six animals in each. The dosages received by the experimental groups were Group-I (Control-Without any sample and/ or negative control), Group-II (Negative control—0.1 mL of 1% carrageenan alone), Group-III (Standard—Indomethacin (20 mg/kg) + 0.1 mL of 1% carrageenan), Group-IV (DoE (125 mg/kg) + (0.1 mL of 1% carrageenan), Group V (DoE (300 mg/kg) + (0.1 mL of 1% carrageenan]. The carrageenan administered as 1% *w*/*v* solution to rats via subcutaneous injection in the left paw-sub plantar region. The paw was stained with temporary ink at the intensity of sideways ankle bone (malleolus) and drenched in mercury up to the mark. The paw volume was measured using Digital Plethysmometer at 0, 1, 2, 3, 4, 5, and 6 h after carrageenan injection. The differentiation between initial and succeeding reading provides the approximate edema volume.

#### 2.3.3. Anti-Cancer Study

For this study, the animals were segregated into seven groups with six animals/group female Sprague Dawley (SD). The experimental groups were assigned as: Group I: control (without any test samples or negative sample); Group II: BaP only (negative control); Group III: BaP + cisplastin (10 mg/kg of SD rat’s body weight); Group IV: DoE (100 mg/kg) + BaP (10 mg/kg); Group V: DoE (150 mg/kg) + BaP (10 mg/kg); Group VI: DoE (200 mg/kg) + BaP (10 mg/kg); Group VII: DoE (250 mg/kg) + BaP (10 mg/kg). The cervical cancer was induced in SD rats using BaP [Benzo (a) pyrene] dissolved in corn oil. The SD rats are the best animal model for studying the anti-cancer evaluation especially for the investigation of reproductive tract cancers. The BaP was finely dissolved in 0.2% of corn oil and administered to SD rats orally and similarly the test samples were put into SD rats orally using specially designed needle; but the cisplastin alone was administered intravenously. The BaP was given to SD rats at an interval of 4 days for about 8 weeks; cisplastin was injected twice a week to Group II animals for a period of 8 weeks and the DoE samples were administered to experimental grouped animals at an interval of four days for a period of 8 weeks. After the stipulated weeks, two SD rats were randomly selected from each group, sacrificed and analyzed for histopathological studies only as described in previously. The hematological and biochemical parameters for each tested group were analyzed from the blood samples withdrawn from three animals selected randomly in each group (*n* = 3).

Further the blood and tissue samples were analyzed for serum tumor markers carcinoembryonic antigen (CEA), gamma-glutamyltransferase (GGT) according to the method of He et al. [31]. The antioxidants and membrane bound enzymes in treated animal samples such as superoxide dismutase (SOD) [32], catalase (CAT), glutathione peroxidase (GPx), total reduced GSH, lipid peroxidation (LPO), and Na^+^K^+^ adenosine triphosphatase [ATPase], Ca^+^ ATPase and Mg+ ATPase were estimated, respectively [33].

### 2.4. Statistical Analysis

The experiments conducted in this investigation were analyzed using statistical tools such as MS Excel and Origin software (OriginPro 2016, Origin Lab Corporation, Northampton, MA, USA). All the values represented in the graphs were mean with standard deviation. The ANOVA significance were depicted in different letters on the respective groups and experiments; the statistical significance was determined using Tukey HSD (honestly significant difference) test.

## 3. Results

### 3.1. Anti-Angiogenic Study by HET-CAM Analysis

**Figure 1:**
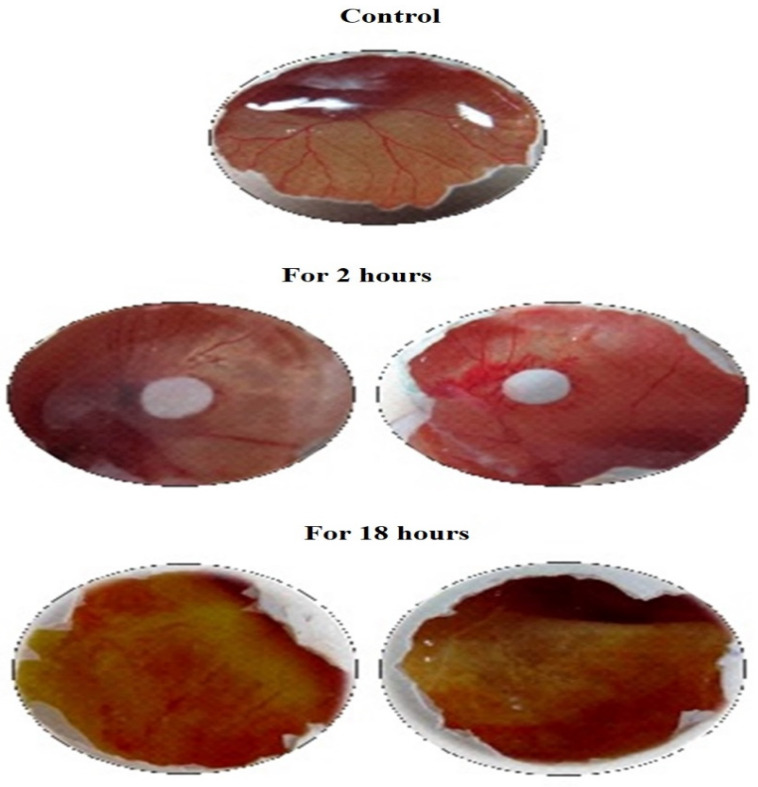
Anti-angiogenic analysis of dimer of epicatechin on egg by HET-CAM test.

The CAM assay is a perceptive, easily feasible, and economy in vivo check for enquiries of the anti-angiogenic promise of individual compounds. The compound DoE exhibited a promising anti-angiogenesis by the inhibition of angiogenesis at an interval of 2 h and 18 h and the inhibition percentage was 72.08% and 91.62, respectively (Table 1 and Figure 1). The solvent acetone explored its potentiality by inhibiting 18.8% and 27.27% for 2 h and 18 h, respectively. This evinced that the DoE has the good anti-angiogenic potentiality which could be explored as anticancer agents.

### 3.2. Animal Studies

#### 3.2.1. Acute Toxicity Analysis in Albino Mice

Acute toxicity is the adverse change(s) which occurs instantly or during a short time within 24 h of administration following a single or short period of exposure to the metabolite. The adverse effect may be any type of effect resulting in functional impairment and/or biochemical lesions which affects the normal activity of the whole organism or specifically disabling a single/multiple organ(s). For this study, the various dosage levels (2 g (Gp II), 1.75 g (Gp III), 1.5 g (Gp IV), 1.25 g (Gp V), 1 g (Gp VI), 0.75 g (Gp VII), 0.5 g (Gp VIII), 0.35 g (Gp IX), 0.25 g (Gp X), and 0.1 g (Gp XI) per Kg of mice, based on the OECD 423 guidelines) were fixed to determine the toxicity of the purified compound DoE in mice models (Supplementary Table S1a–j) and the control group animals were fixed in group I. Through this analysis the effective dosage for the survival of the animal was 1.25 g/kg (Gp V) and the lethal dosage was 1.5 g/kg (Gp IV). Above 1.5 g/kg of the DoE produced hyper sensitivity, righting reflex, tremors and convulsions leading to the death of the animal.

**Figure 2:**
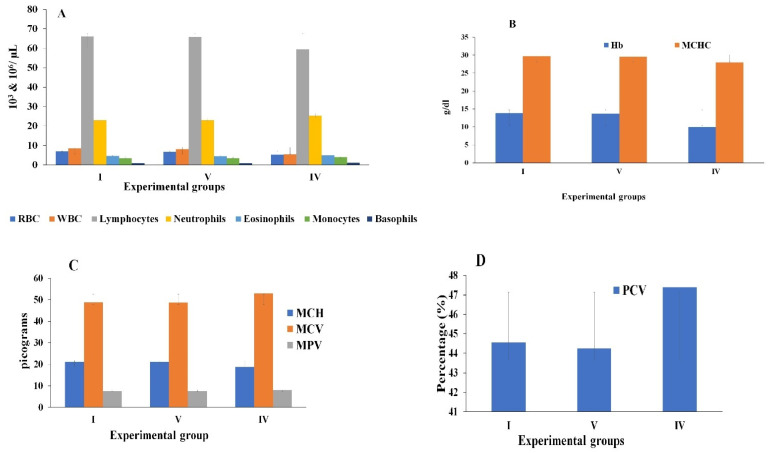
Analysis of hematological parameters in treated and control Albino mice. The data presented as mean value ± SE (*n* = 3). Group I: Control; Group V: survival (1250 mg/kg); Group IV: lethal (1500 mg/kg). (**A**) Blood hematological analysis (RBC: Red Blood Corpuscles; WBC: White Blood Corpuscles); (**B**) Hb: Hemoglobin; MCHC: Mean Corpuscular Hemoglobin Concentration; (**C**) MCH: Mean Corpuscular Hemoglobin; MCV: Mean Corpuscular Volume; and MPV: Mean Platelet Volume; (**D**) Packed Cell Volume (PCCV) analysis.

The hematological parameters were evaluated for the survival and lethal dosage grouped animals compared with control group (Figure 2A–D). The RBC count was similar to the control group whereas the count was low in lethal group. The WBC count was highest in lethal group than control group, this is because the lethality has been identified by the immune system and thereby the cells arrives for defensive mechanism in order to eliminate the excessive toxicity out the body. The granulocytes (neutrophils, eosinophils and basophils) and agranulocytes (lymphocytes and monocytes) count of the blood sample was similar in both survival and control groups but there was a slight variation in the lethal group and this indicated the severity of overdose to the body weight. The hemoglobin, MCH and MCHC count were observed to be decreased in the lethal grouped animals when compared to survival and control grouped animals. The MCV and MPV were found to be similar in both control and group V animals but the volume was higher in lethal group. The packed cell volume (PCV) was higher than in group V and control group animals. This conclude that the DoE dose above 1.25 g/kg affects the blood cells and the total corpuscular volume.

Similarly, the biochemical parameters were evaluated for the control, survival, and lethal group (Group I, V, and IV) and results were depicted in Table 2. The biochemical such as AST, ALT, ALP, bilirubin, and total proteins were found to be rising significant in an order for survival and lethal group when compared to control group. This implicit that the compound DoE is an effective drug which does not alter the biochemical and hematological parameters in the limited dosage.

**Figure 3:**
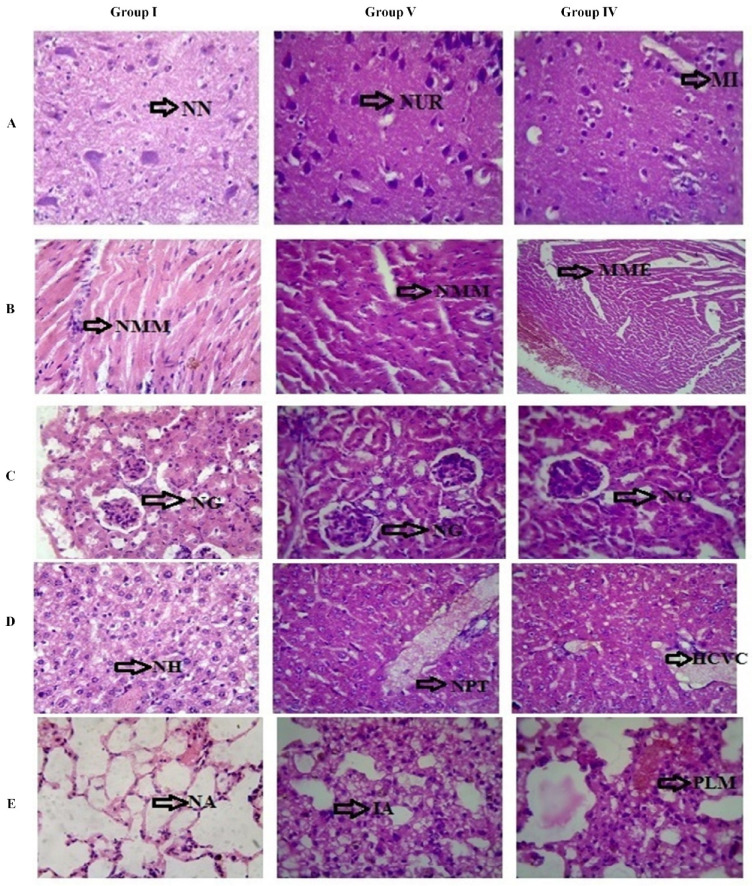
Histopathological analysis of acute toxicity studies. Group I: Control; Group V: survival (1250 mg/kg); Group IV: lethal (1500 mg/kg). (**A**) Brain section; (**B**) Heart section; (**C**) Kidney section; (**D**) Liver section; (**E**) Lung section; NN: Normal Neurons; NUR: Neurons Unremarkable; NMM: Normal Myocardium Myocytes; MME: Myocytes Mild Edema; NG: Normal Glomerulus; NH: Normal Hepatocytes; NPT: Normal Portal Tract; HCVC: Hepatocyte Central Vein Congestion; NA: Normal Alveoli; IA: Interconnected Alveoli; PLM: Pigment Laden Macrophages.

A study has also been performed to check out the reason for lethality and survival in the above said dosages (Group IV and V) along with the control (Group I) through the histopathology analysis of five important organs such as liver, lungs, heart, brain, and kidneys. All the organs except the lung tissue were observed to be in normal cellular/tissue morphology (Figure 3A–E) whereas the tissues from lungs of animals administered with lethal dose exhibited interconnected alveoli and this might be the prime reason behind the lethality of the animals.

#### 3.2.2. Anti-Inflammatory Study

**Figure 4:**
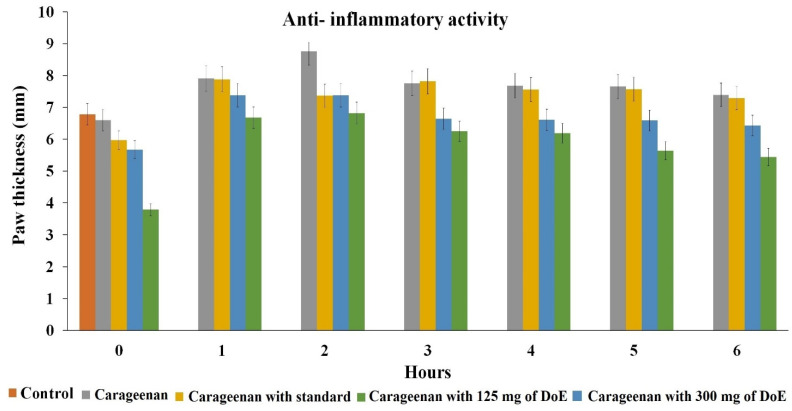
Anti-inflammatory analysis of dimer of epicatechin in Wistar rats. The data presented as mean value ± SE (*n* = 3). The model was significant with *p* < 0.05.

**Figure 5:**
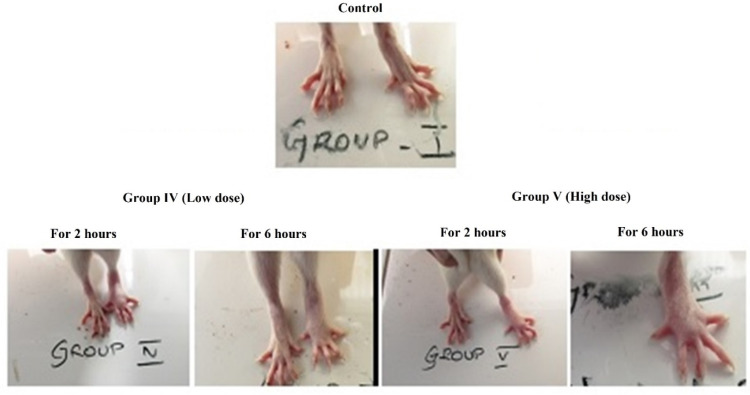
Anti-inflammatory response of dimer of epicatechin (DoE) on Wistar rats.

From the earlier evaluation, the DoE was confirmed to be a potent free radical scavenger, as it has the capacity to reduce the inflammatory response of inflammation/ induced paw edema and this was assessed through carrageenan induced anti-inflammatory Wistar rat models. The investigation results revealed that the DoE is an effective metabolite in reducing the inflammation induced by carrageenan (Figure 4 and Figure 5). The animals developed the maximum inflammatory response at 3 h of induction and after 3 h, the inflammation started to reduce gradually. The percentage of inhibition was found to be 41.09% (300 mg/kg) which was found to be twice than the standard drug indomethacin (20.17%) used. This proved the DoE was an efficient anti-inflammatory drug.

#### 3.2.3. Anti-Cancer Study

The excessive production of reactive oxygen species (ROS) in the cervical tissues/cells might damage and/or induces the rapid proliferation of cells and leads to cervical neoplasia, lesions, and carcinoma. There may be several reasons for the onset of cervical cancer such as human papilloma virus (HPV) infections, unhealthy lifestyle, etc., but the oxidative stress with the increased redox potentials is one the major reason for the carcinoma. The cervical tissues under severe alteration in the redox oxidative stress leads to change in the gene expression that might induces the cellular proliferation in a rapid state. The cervical cancer can be easily reduced or treated with the several medicines including traditional and food diet. One such potent therapeutic/medicine is polyphenols from natural sources containing the anti-cancer properties and there are several researches explicated the therapeutic nature of the polyphenols against cervical cancer. In this study the inhibition of cervical carcinoma’s malignant stage was effectively treated by the phenolic product-DoE.

**Figure 6:**
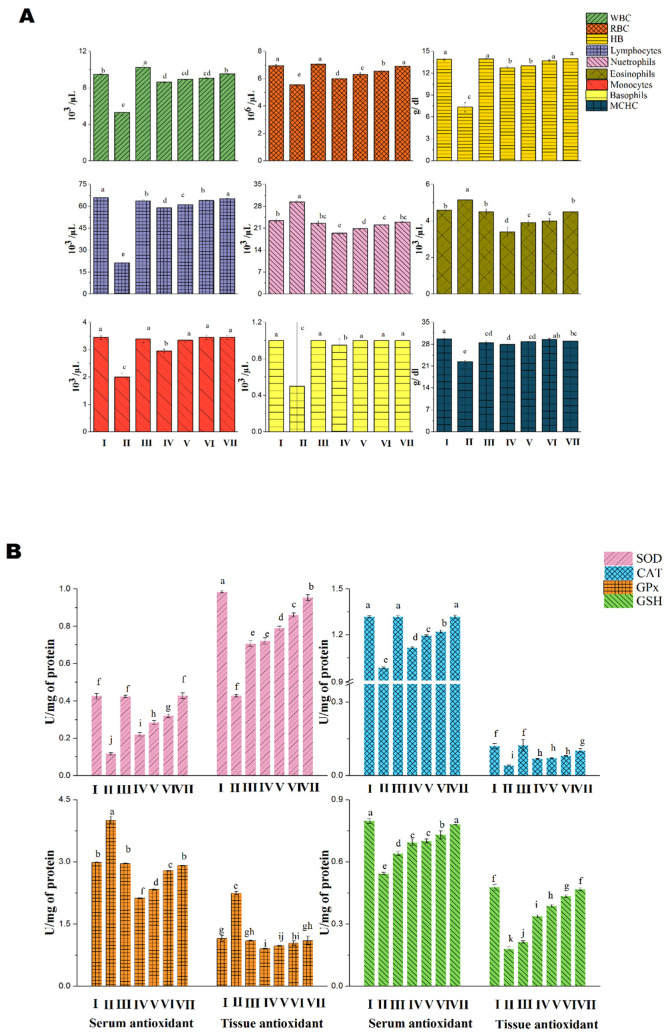

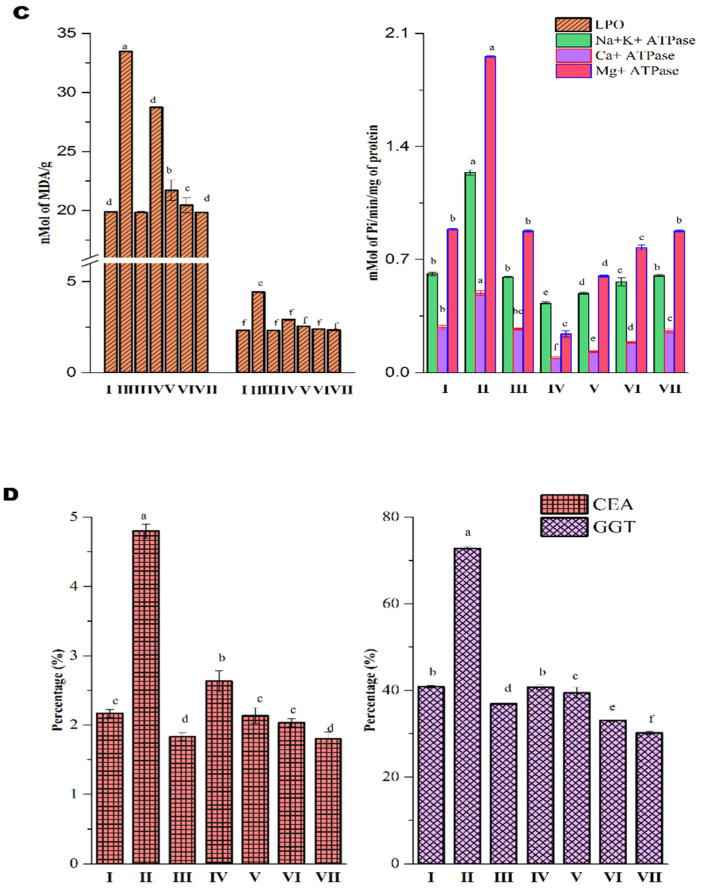
Determination of hematological, antioxidants, lipid peroxidation (LPO), membrane bound enzymes and tumor markers in control and treated Sprague Dawley (SD) animals. (**A**) Hematological parameters; (**B**) Antioxidants (CAT, SOD, GPx and GSH) in serum and tissues; (**C**) LPO and membrane bound enzymes in serum; (**D**) carcinogenic markers—CEA and GGT. All the graphs (**A**–**D**) are represented in mean ± standard deviation (*n* = 3). Different alphabets (a–j) indicate significant differences among treatments (*p* < 0.05)—based upon the differences between the experimental groups) according to Tukey’s HSD test.

**Figure 7:**
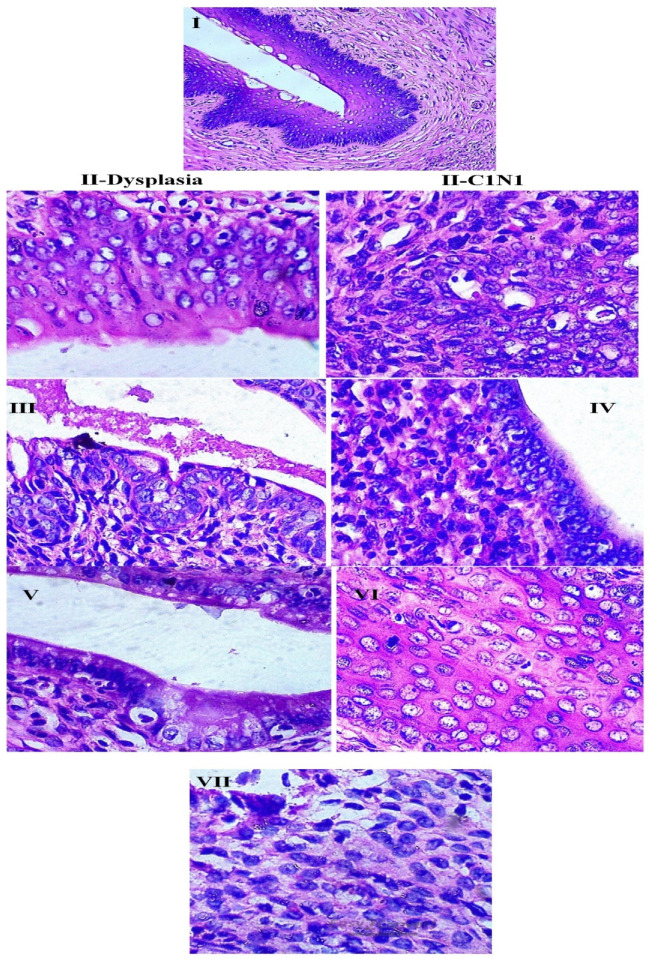
Histopathology of cervical tissues for control and drug(s) treated SD animals. SD rats from each group was randomly selected. **I**–**VII**: Experimental groups; BaP: Benzo (a) pyrene; DoE: Dimer of Epicatechin; CIN1: Cervical intraepithelial neoplasia 1; IED-N: Intraepithelial cells disturbance-neoplasia; SEJ: squamous columnar epithelial junction.

The blood cells such as RBC, WBC, agranulocytes, and granulocytes count were found to be normal in control, cisplatin treated, and DoE treated group of animals (Figure 6A) and the experiments were found to significant. There was a lowest count of blood cells in the group IV but when there was a steady increase in the dosage of DoE, the blood cells also reached the normal range. The antioxidants in the serum such as SOD, CAT, GPx, and GSH was similar in Group I, III, and VII; this exhibited the standard drug cisplatin and DoE has the similar therapeutic index (Figure 6B). The dose of 250 mg DoE has a good potential in the reduction of elevated oxidative stress and redox potentials, which could further aids in the normal cell functioning and apoptosis. Similarly, the antioxidants in the cervical tissues also found to be normal range in control, cisplatin and DoE treated animals (Figure 6B). The ANOVA for the groups tested for antioxidants presence in the serum and tissues were accounted as significant (*p* < 0.05). The LPO was measured by the production of MDA as an important oxidant which damages the DNA; this MDA is formed as an end product of LPO when the ROS reacts with the PUFA. The LPO in serum and tissues was found to higher in group II whereas there was a significant decrease when animals treated with cisplatin and DoE in a dose-dependent manner (Figure 6C). The count of membrane bound enzymes for the control and treated grouped animals were exhibited in the Figure 6C and there was a similar range found in both DoE and cisplatin treated animals; the test was significant with the p value of less than 0.05. The carcinogenic marker for the CEA and GGT for the control and treated groups were given in Figure 6D. The histopathological analysis of the cervical tissues for control and treated grouped animals were depicted in Figure 7.

## 4. Discussion

Cervical cancer is one of the biggest threats in the world among women and it is the second most common cancer [32,34]. The highest causative reason is oxidative stress, and this has to be managed properly otherwise the excessive production of ROS in the cell alters the gene expression and transcriptional factors leading to highest rate of cell progression. This is mainly caused by the poor presence of antioxidants in the cell and increased rate of ROS in the aerobic cells [35]. The ROS are mainly formed during the oxidative phosphorylation in the mitochondria where the molecular oxygen (O_2_) changes into singlet O_2_ or H_2_O_2_ and this is initiated by the several processes such as UV or radiation exposures, chemical and mechanical stresses, inflammatory reactions and repeated microbial infections [36,37]. The increased progression of ROS or oxidative stress in the cervical tissues further alters the properties of DNA and progresses as cervical neoplasia, cervical tumor, and cervical cancer. The reduction of ROS and prevention of ROS can be easily succeeded with the aid of polyphenols and flavonoids which are effective antioxidants. Currently, several research focused to extract the polyphenols and flavonoids from endophytic fungi and the metabolites were explored as anti-cancer metabolites in medicinal fields [3,35].

The current investigation has produced and purified a potential polyphenolic DoE from a prospective endophytic fungus *C. australiensis* FC2AP and the DoE belongs to the class of chemical family, flavonoids. The flavonoids have therapeutic metabolites or products exhibiting the antimicrobial, antioxidant, antiviral, antiparasitic, and anti-cancer properties. Angiogenesis in tumor or tumor cells is a malignant stage and this could be minimized or prevented by the implementation of polyphenols and flavonoids. The preliminary assessment of inhibiting the angiogenesis process was successfully carried out through HET-CAM analysis in chick’s embryonated eggs and the DoE inhibited and slowed down the formation of blood vessels in the embryo. The tumor progression at various sites are initiated by the onset of the angiogenesis process—one of the characteristic in cancer cells, and thus the cancer cells are carried over the blood stream further invading any organs/sites in the body; this stage is called metastasis. Angiogenesis in tumors/carcinoma is an essential or vital component in cancer growth and propagation, and an important target for investigations related to its therapeutic use. It is significant to consider that tumor vascularization not only supply nutrients to tumors/tumor invasive areas, but also controls the physiopathology, and consequently its growth, metastasis in tumor [38]. Thus, inhibiting tumor angiogenesis may halt the tumor growth and decrease metastatic potential of tumors. Yildiz et al. [39] reported the inhibition of angiogenic effects using few anticancer products such as imatinib, diltiazem and bevacizumab. Pyripyropene A and hypocrimins (A and B) isolated from *Aspergillus niger* and *Hypocrea vinosa,* respectively, have good anti-angiogenic properties [40]; Similarly a synthetic analogue pinabulin was derived from *Aspergillus* sp. and fumagillin from *A. fumigatus* have been exhibiting the anti-angiogenic effects.

Further the metabolite DoE was subjected to in vivo studies (animal models) in a sequential manner starting with the dosage fixing through acute toxicity analysis. Usually, any drug of 2 g/kg body weight of the animal is a lethal dose and in this investigation the animals survived at the 75% of maximum dosage level. The highest survival rate of the animals in this toxicity analysis was due to the DoE which is a polyphenol compound explicating the antioxidant properties. The catechins and epicatechins contains the properties such as antioxidants, anti-angiogenic, anti-proliferative, proapoptotic, anti-metastatic, and cell cycle perturbation [35,41,42,43]. The hematological and biochemical parameters are the most sensitive parameters for the analysis of toxicity in the animals and human; moreover, the determination assess the pathophysiological conditions in the blood stream [44,45].

Inflammation is the major problem in the tumor progression because the long-term inflammation in a site of the body could eventually leads to the formation of cancer. Balkwill and Mantovani [46] found out there was a correlation between the inflammation and cancer, and this condition is a predisposition of cancer. The inflammation may be due to prolonged infections by the microbial invasions and if the infection were untreated in the specific cervical tissues that could eventually leads to the neoplasia or cervical carcinoma. Moreover, the inflammation promotes the malignant tumor growth or progression in the surrounding tissues of the inflammation area and this inflammation is a chronic and unregulated process [47]. The alteration in the gene expression in the inflammation surrounding cells/tissues is induced by the production of ROS or nitrogen species in the inflammation site. As a result of the alteration in the genetic factors, the inflammation plays a crucial role in initiation of tumor, malignancy and metastatic processes [48]. Various factors like bradykinins, histamines, serotonine, prostaglandins, oxidative stress, and free radicals such as superoxide anion, NO, and hydroxyl radicals are responsible for inflammation [49,50]. In this investigation, 1/10th of the effective dose was taken for the anti- inflammatory analysis in carrageenan-induced Wistar rats. Carrageenan-induced paw edema is a widely used test to determine the anti-inflammatory activity [51]. The DoE reduced the inflammation within few hours and this explicit the DoE has the capability in decreasing vascular permeability and cellular infiltration where the accumulation of leukocytes, proteins and fluids are minimized by the respective DoE dosage levels. The given DoE has the capacity in clearing out the debris and scavenges the ROS and/or free radicals in the injured or inflamed site which prevents the damages or alteration in the DNA of the cells [52].

With the results of anti-inflammatory responses, the cervical carcinoma analysis was assessed in BaP induced cancer Sprague Dawley rats. The cancer markers, antioxidants in serum and tissues, LPO in serum and tissues were found to be normal in DoE treated animals and the count were similar to the control group animals. Moreover, the DoE treated grouped results exhibited good survival rate and the hematological parameters were approximate with the blood count of standard drug: cisplatin treated animals. The endophytic fungal metabolites are very efficient in treating various types of cancer and there were several researches found various types of anti-tumor metabolites [53,54]. He et al. [4] identified that the anti-cancer therapeutic secondary metabolite from an endophytic fungus *Gingko biloba* and the metabolite exhibited effective activity against cervical cancer in mice. The metabolites such as catechins, epicatechins, epigallocatechin gallate, and epigallocatechin are efficient antioxidants, and these are highly present in the green tea [55]. Shibuya et al. [56] reported that the presence of catechins and epicatechins produced by the endophytic fungus *Diaporthe* sp. had been transformed into dihydroxyflavan derivatives and the endophyte was isolated from the tea plant. Most of the catechins and epicatechin derivatives were extracted from the tea plants but with respect to this investigation, the catechins and epicatechins were present in *A. marmelos* tree and this is the reason the endophyte isolated from this host tree produced the same metabolites [19].

The epicatechins and its derivatives have a significant role in treating the cervical cancer by minimizing the cell proliferation in several ways, such as (a) induction of cancerous cell cycle arrest; (b) modulating cancer cell growth; (c) depolymerization of cellular microtubules and inhibition of tubulin assembly; (d) arresting the angiogenesis in cancer cells; and (e) restriction of oncoproteins which has been induced by HPV and oxidative stresses [10]. The epicatechins family contains the properties in scavenging the free radicals produced during the mitochondrial oxidative stress efficiently [57,58]. Several researchers has investigated the cervical cancer therapeutic properties of epicatechins and its derivatives on arresting the cells in G1 phase [59]; inhibition of signal transduction pathways; activation of redox sensitive transcriptional factors such as AP1 and NF-κB in the cell lines [60,61]; inhibition of EGFR signaling which is a beginning kinase in EGF cascades and activation of ERK1/2 and AKT activities [60]; inhibition of telomerase activity in the progression of cervical cancer lesions [62,63,64]; inhibition of proteasomal activities in HeLa cell line; and suppression of mRNA, ERα and aromatase (protein expression) as a result, the cervical cancer cells enters the apoptotic stage [65]. The telomerase activity in the endocervical and ectocervical cancer cells in human was inhibited by the therapeutic drug EGCG [63]. Moreover, in cancer cells, there is a vital role of microtubules majorly in signaling process, proliferation, and metastasis (migration) processes in cervical cancer cells; therefore, the microtubule and tubulin are decisive targets for anti-cancer medicines/drugs. The investigation of Chakraborty et al. [66] has studied inhibition of HeLa cells’ proliferation by the implementation of Epicatechins and its derivative and the proliferation of the cancer cells were formed through depolymerizing cellular microtubule and restraining tubulin assembly. Hence, the DoE of current investigation has explicated its maximized anti-cervical cancer activity which was like the epicatechins and its derivatives as stated in the foresaid research. The apoptosis on the HeLa cell lines was effectively implemented by the DoE; the further studies will be explored to determine the inhibition of proliferation in cancer cells and also to analyze the metabolic mechanisms between DoE and cancer cells.

## 5. Conclusions

In summary, the results obtained in the present study indicated that the metabolite DoE purified from the CPME of *C. australiensis* explored that their potentiality at the minimum concentration. The DoE exhibited its therapeutic properties on cervical cancer cells in minimum dosage; further the carrageenan-induced inflammation on animal models were significantly reduced by the implication of the DoE. This proved that the DoE explicated its therapeutic index towards the treatment of cervical cancer in animal models. Thus, the application of DoE in the cancer therapeutics will reduce the side effects of chemotherapy and aims in curing the cancer. Future research is needed to determine the role of DoE on other tumor cells and the molecular mechanisms against anticancer studies for further development of natural anticancer substances.

## Figures and Tables

**Table 1:** Anti-angiogenesis effect of dimer of epicatechin in Hen’s Egg Test on Chorio Allantoic Membrane (HET-CAM) test.

Sample	For 2 h			For 18 h		
	No. of Vessels in Untreated CAM	No. of Vessels in Treated CAM	Inhibition (%)	No. of Vessels in Untreated CAM	No. of Vessels in Treated CAM	Inhibition (%)
Negative Control (0.9% NaCl)	09	09	0	09	09	0
Sample-1	12	04	66.6	12	01	91.67
Sample-1	13	03	76.92	13	01	92.3
Sample-1	11	03	72.72	11	01	90.9
Positive Control (1N NaOH)	08	02	75.0	08	0	100
Acetone	11	09	18.18	11	08	27.27

NaCl: Sodium chloride; NaOH: Sodium hydroxide; CAM: Chorio Allantoic Membrane; Sample 1: 200 µL of DoE.

**Table 2:** Biochemical parameters of Albino mice in acute toxicity study.

S. No.	Biochemical Markers	Group I	Group V	Group IV
1	ALP (IU/L)	118.02 ± 0.07	111.22 ± 0.98	178.45 ± 0.98
2	ALT (IU/L)	52.8 ± 0.41	51.18 ± 0.82	98.4 ± 0.82
3	AST (IU/L)	69.16 ± 0.49	70.24 ± 0.61	203.39 ± 0.52
4	Bilirubin (mg/dL)	0.95 ± 0.03	0.92 ± 0.30	6.17 ± 0.05
5	Total protein (mg/dL)	9.4 ± 0.36	9.5 ± 0.24	7.31 ± 0.25

The data presented as mean value ± SE (*n* = 3). Group I: Control; Group V: survival (1250 mg/kg); Group IV: lethal (1500 mg/kg). ALP: Alkaline Phosphatase Test; ALT: Alanine Amino Transferase; AST: Aspartate Amino Transferase; IU/L: International Units per Liter.

## Data Availability

The data presented in this study are available on request from the corresponding author.

## Supplementary Materials

The following are available online, Table S1: Toxicological evaluations of purified compound DoE.

## Sample Availability

Samples of the compounds are not available from the authors.
