# Supplementary material for: Evaluation of Dimer of Epicatechin from an Endophytic Fungus Curvularia australiensis FC2AP on Acute Toxicity Levels, Anti-Inflammatory and Anti-Cervical Cancer Activity in Animal Models
Source: Molecules. 2021 Jan 27;26(3):654. doi: 10.3390/molecules26030654 (PMC7866062; doi:10.3390/molecules26030654)
Supplement: Supplementary file 1 [file molecules-26-00654-s001.pdf]

Table. S1. Toxicological evaluations of purified compound DoE

a. Dose: Compound DoE (2 g/kg)

Observation	Head		Body		Tail		Head & Body		Body &tail		No Marking	
	Before	After	Before	After	Before	After	Before	After	Before	After	Before	after
Alertness	N	A	N	A	N	A	N	A	N	A	N	A
Grooming	A	A	A	A	A	A	A	A	A	A	A	A
Touch response	P	A	P	A	P	A	P	A	P	A	P	A
Torch response	P	P	P	A	P	A	P	A	P	A	P	A
Pain response	A	P	A	A	A	A	A	A	A	A	A	A
Tremors	A	P	A	P	A	P	A	P	A	P	A	P
Convulsions	A	P	A	P	A	P	A	P	A	P	A	P
Righting reflex	A	P	A	P	A	P	A	P	A	P	A	P
Gripping strength	N	A	N	A	N	A	N	A	N	A	N	A
Pinna reflex	N	A	N	A	N	A	N	A	N	A	N	A
Corneal reflex	N	N	N	N	N	N	N	N	N	N	N	N
Pupils	N	N	N	N	N	N	N	N	N	N	N	N
Urination	N	P	N	P	N	P	N	P	N	P	N	P
Salivation	A	P	A	P	A	P	A	P	A	P	A	P
Skin colour	N	P	N	P	N	P	N	P	N	P	N	P
Lacrimation	A	P	A	P	A	P	A	P	A	P	A	P
Hyperactivity	N	P	N	P	N	P	N	P	N	P	N	P

N – Normal; A – Absent; P - Present

b. Dose: Compound DoE (1.75 g/kg)

Observation	Head		Body		Tail		Head & Body		Body &tail		No Marking	
	Before	After	Before	After	Before	After	Before	After	Before	After	Before	after
Alertness	N	A	N	A	N	A	N	A	N	A	N	A
Grooming	A	A	A	A	A	A	A	A	A	A	A	A
Touch response	P	A	P	A	P	A	P	A	P	A	P	A
Torch response	P	A	P	A	P	A	P	A	P	A	P	A
Pain response	A	A	A	A	A	A	A	A	A	A	A	A
Tremors	A	P	A	P	A	P	A	P	A	P	A	P
Convulsions	A	P	A	P	A	P	A	P	A	P	A	P
Righting reflex	A	P	A	P	A	P	A	P	A	P	A	P
Gripping strength	N	A	N	A	N	A	N	A	N	A	N	A
Pinna reflex	N	A	N	A	N	A	N	A	N	A	N	A
Corneal reflex	N	N	N	N	N	N	N	N	N	N	N	N
Pupils	N	N	N	N	N	N	N	N	N	N	N	N
Urination	N	P	N	P	N	P	N	P	N	P	N	P
Salivation	A	P	A	P	A	P	A	P	A	P	A	P
Skin colour	N	P	N	P	N	P	N	P	N	P	N	P
Lacrimation	A	P	A	P	A	P	A	P	A	P	A	P
Hyperactivity	N	P	N	P	N	P	N	P	N	P	N	P

N – Normal; A – Absent; P – Present

c. Dose: Compound DoE (1.5 g/kg)

Observation	Head		Body		Tail		Head & Body		Body &tail		No Marking	
	**Before**	**After**	**Before**	**After**	**Before**	**After**	**Before**	**After**	**Before**	**After**	**Before**	**after**
Alertness	N	N	N	N	N	N	N	N	N	N	N	N
Grooming	A	A	A	A	A	A	A	A	A	A	A	A
Touch response	P	P	P	P	P	P	P	P	P	P	P	P
Torch response	P	P	P	P	P	P	P	P	P	P	P	P
Pain response	A	P	A	P	A	P	A	P	A	P	A	P
Tremors	A	P	A	P	A	P	A	P	A	P	A	P
Convulsions	A	P	A	P	A	P	A	P	A	P	A	P
Righting reflex	A	P	A	P	A	P	A	P	A	P	A	P
Gripping strength	N	A	N	A	N	A	N	A	N	A	N	A
Pinna reflex	N	A	N	A	N	A	N	A	N	A	N	A
Corneal reflex	N	N	N	N	N	N	N	N	N	N	N	N
Pupils	N	N	N	N	N	N	N	N	N	N	N	N
Urination	N	P	N	P	N	P	N	P	N	P	N	P
Salivation	A	P	A	P	A	P	A	P	A	P	A	P
Skin colour	N	P	N	P	N	P	N	P	N	P	N	P
Lacrimation	A	P	A	P	A	P	A	P	A	P	A	P
Hyperactivity	N	P	N	P	N	P	N	P	N	P	N	P

N – Normal; A – Absent; P - Present

d. Dose: Compound DoE (1.25 g/kg)

Observation	Head		Body		Tail		Head & Body		Body &tail		No Marking	
	Before	After	Before	After	Before	After	Before	After	Before	After	Before	after
Alertness	N	N	N	N	N	N	N	N	N	N	N	N
Grooming	A	A	A	A	A	A	A	A	A	A	A	A
Touch response	P	P	P	P	P	P	P	P	P	P	P	P
Torch response	P	P	P	P	P	P	P	P	P	P	P	P
Pain response	A	P	A	P	A	P	A	P	A	P	A	P
Tremors	A	P	A	P	A	P	A	P	A	P	A	P
Convulsions	A	P	A	P	A	P	A	P	A	P	A	P
Righting reflex	A	A	A	A	A	A	A	A	A	A	A	A
Gripping strength	N	A	N	A	N	A	N	A	N	A	N	A
Pinna reflex	N	A	N	A	N	A	N	A	N	A	N	A
Corneal reflex	N	N	N	N	N	N	N	N	N	N	N	N
Pupils	N	N	N	N	N	N	N	N	N	N	N	N
Urination	N	N	N	N	N	N	N	N	N	N	N	N
Salivation	A	N	A	N	A	N	A	N	A	N	A	N
Skin colour	N	N	N	N	N	N	N	N	N	N	N	N
Lacrimation	A	N	A	N	A	N	A	N	A	N	A	N
Hyperactivity	N	A	N	A	N	A	N	A	N	A	N	A

N – Normal; A – Absent; P - Present

e. **Dose: Compound DoE (1 g/kg)**

Observation	Head		Body		Tail		Head & Body		Body &tail		No Marking	
	**Before**	**After**	**Before**	**After**	**Before**	**After**	**Before**	**After**	**Before**	**After**	**Before**	**after**
Alertness	N	N	N	N	N	N	N	N	N	N	N	N
Grooming	A	A	A	A	A	A	A	A	A	A	A	A
Touch response	P	P	P	P	P	P	P	P	P	P	P	P
Torch response	P	P	P	P	P	P	P	P	P	P	P	P
Pain response	A	P	A	P	A	P	A	P	A	P	A	P
Tremors	A	P	A	P	A	P	A	P	A	P	A	P
Convulsions	A	P	A	P	A	P	A	P	A	P	A	P
Righting reflex	A	A	A	A	A	A	A	A	A	A	A	A
Gripping strength	N	A	N	A	N	A	N	A	N	A	N	A
Pinna reflex	N	A	N	A	N	A	N	A	N	A	N	A
Corneal reflex	N	N	N	N	N	N	N	N	N	N	N	N
Pupils	N	N	N	N	N	N	N	N	N	N	N	N
Urination	N	N	N	N	N	N	N	N	N	N	N	N
Salivation	A	A	A	A	A	A	A	A	A	A	A	A
Skin colour	N	N	N	N	N	N	N	N	N	N	N	N
Lacrimation	A	A	A	A	A	A	A	A	A	A	A	A
Hyperactivity	N	N	N	N	N	N	N	N	N	N	N	N

N – Normal; A – Absent; P - Present

f. Dose: Compound DoE (0.75 g/kg)

Observation	Head		Body		Tail		Head & Body		Body &tail		No Marking	
	**Before**	**After**	**Before**	**After**	**Before**	**After**	**Before**	**After**	**Before**	**After**	**Before**	**after**
Alertness	N	N	N	N	N	N	N	N	N	N	N	N
Grooming	A	A	A	A	A	A	A	A	A	A	A	A
Touch response	P	P	P	P	P	P	P	P	P	P	P	P
Torch response	P	P	P	P	P	P	P	P	P	P	P	P
Pain response	A	A	A	A	A	A	A	A	A	A	A	A
Tremors	A	A	A	A	A	A	A	A	A	A	A	A
Convulsions	A	A	A	A	A	A	A	A	A	A	A	A
Righting reflex	A	A	A	A	A	A	A	A	A	A	A	A
Gripping strength	N	N	N	N	N	N	N	N	N	N	N	N
Pinna reflex	N	N	N	N	N	N	N	N	N	N	N	N
Corneal reflex	N	N	N	N	N	N	N	N	N	N	N	N
Pupils	N	N	N	N	N	N	N	N	N	N	N	N
Urination	N	N	N	N	N	N	N	N	N	N	N	N
Salivation	A	A	A	A	A	A	A	A	A	A	A	A
Skin colour	N	N	N	N	N	N	N	N	N	N	N	N
Lacrimation	A	A	A	A	A	A	A	A	A	A	A	A
Hyperactivity	N	N	N	N	N	N	N	N	N	N	N	N

N – Normal; A – Absent; P - Present

g. Dose: Compound DoE (0.5 g/kg)

Observation	Head		Body		Tail		Head & Body		Body &tail		No Marking	
	Before	After	Before	After	Before	After	Before	After	Before	After	Before	after
Alertness	N	N	N	N	N	N	N	N	N	N	N	N
Grooming	A	A	A	A	A	A	A	A	A	A	A	A
Touch response	P	P	P	P	P	P	P	P	P	P	P	P
Torch response	P	P	P	P	P	P	P	P	P	P	P	P
Pain response	A	A	A	A	A	A	A	A	A	A	A	A
Tremors	A	A	A	A	A	A	A	A	A	A	A	A
Convulsions	A	A	A	A	A	A	A	A	A	A	A	A
Righting reflex	A	A	A	A	A	A	A	A	A	A	A	A
Gripping strength	N	N	N	N	N	N	N	N	N	N	N	N
Pinna reflex	N	N	N	N	N	N	N	N	N	N	N	N
Corneal reflex	N	N	N	N	N	N	N	N	N	N	N	N
Pupils	N	N	N	N	N	N	N	N	N	N	N	N
Urination	N	N	N	N	N	N	N	N	N	N	N	N
Salivation	A	A	A	A	A	A	A	A	A	A	A	A
Skin colour	N	N	N	N	N	N	N	N	N	N	N	N
Lacrimation	A	A	A	A	A	A	A	A	A	A	A	A
Hyperactivity	N	N	N	N	N	N	N	N	N	N	N	N

N – Normal; A – Absent; P - Present

h. Dose: Compound DoE (0.35 g/kg)

Observation	Head		Body		Tail		Head & Body		Body &tail		No Marking	
	Before	After	Before	After	Before	After	Before	After	Before	After	Before	after
Alertness	N	N	N	N	N	N	N	N	N	N	N	N
Grooming	A	A	A	A	A	A	A	A	A	A	A	A
Touch response	P	P	P	P	P	P	P	P	P	P	P	P
Torch response	P	P	P	P	P	P	P	P	P	P	P	P
Pain response	A	A	A	A	A	A	A	A	A	A	A	A
Tremors	A	A	A	A	A	A	A	A	A	A	A	A
Convulsions	A	A	A	A	A	A	A	A	A	A	A	A
Righting reflex	A	A	A	A	A	A	A	A	A	A	A	A
Gripping strength	N	N	N	N	N	N	N	N	N	N	N	N
Pinna reflex	N	N	N	N	N	N	N	N	N	N	N	N
Corneal reflex	N	N	N	N	N	N	N	N	N	N	N	N
Pupils	N	N	N	N	N	N	N	N	N	N	N	N
Urination	N	N	N	N	N	N	N	N	N	N	N	N
Salivation	A	A	A	A	A	A	A	A	A	A	A	A
Skin colour	N	N	N	N	N	N	N	N	N	N	N	N
Lacrimation	A	A	A	A	A	A	A	A	A	A	A	A
Hyperactivity	N	N	N	N	N	N	N	N	N	N	N	N

N – Normal; A – Absent; P - Present

i. **Dose: Compound DoE (0.25 g/kg)**

Observation	Head		Body		Tail		Head & Body		Body &tail		No Marking	
	Before	After	Before	After	Before	After	Before	After	Before	After	Before	after
Alertness	N	N	N	N	N	N	N	N	N	N	N	N
Grooming	A	A	A	A	A	A	A	A	A	A	A	A
Touch response	P	P	P	P	P	P	P	P	P	P	P	P
Torch response	P	P	P	P	P	P	P	P	P	P	P	P
Pain response	A	A	A	A	A	A	A	A	A	A	A	A
Tremors	A	A	A	A	A	A	A	A	A	A	A	A
Convulsions	A	A	A	A	A	A	A	A	A	A	A	A
Righting reflex	A	A	A	A	A	A	A	A	A	A	A	A
Gripping strength	N	N	N	N	N	N	N	N	N	N	N	N
Pinna reflex	N	N	N	N	N	N	N	N	N	N	N	N
Corneal reflex	N	N	N	N	N	N	N	N	N	N	N	N
Pupils	N	N	N	N	N	N	N	N	N	N	N	N
Urination	N	N	N	N	N	N	N	N	N	N	N	N
Salivation	A	A	A	A	A	A	A	A	A	A	A	A
Skin colour	N	N	N	N	N	N	N	N	N	N	N	N
Lacrimation	A	A	A	A	A	A	A	A	A	A	A	A
Hyperactivity	N	N	N	N	N	N	N	N	N	N	N	N

N – Normal; A – Absent; P - Present

j. Dose: Compound DoE (0.1 g/kg)

Observation	Head		Body		Tail		Head & Body		Body &tail		No Marking	
	**Before**	**After**	**Before**	**After**	**Before**	**After**	**Before**	**After**	**Before**	**After**	**Before**	**after**
Alertness	N	N	N	N	N	N	N	N	N	N	N	N
Grooming	A	A	A	A	A	A	A	A	A	A	A	A
Touch response	P	P	P	P	P	P	P	P	P	P	P	P
Torch response	P	P	P	P	P	P	P	P	P	P	P	P
Pain response	A	A	A	A	A	A	A	A	A	A	A	A
Tremors	A	A	A	A	A	A	A	A	A	A	A	A
Convulsions	A	A	A	A	A	A	A	A	A	A	A	A
Righting reflex	A	A	A	A	A	A	A	A	A	A	A	A
Gripping strength	N	N	N	N	N	N	N	N	N	N	N	N
Pinna reflex	N	N	N	N	N	N	N	N	N	N	N	N
Corneal reflex	N	N	N	N	N	N	N	N	N	N	N	N
Pupils	N	N	N	N	N	N	N	N	N	N	N	N
Urination	N	N	N	N	N	N	N	N	N	N	N	N
Salivation	A	A	A	A	A	A	A	A	A	A	A	A
Skin colour	N	N	N	N	N	N	N	N	N	N	N	N
Lacrimation	A	A	A	A	A	A	A	A	A	A	A	A
Hyperactivity	N	N	N	N	N	N	N	N	N	N	N	N

N – Normal; A – Absent; P - Present
